# The genetic basis of hybrid male sterility in sympatric *Primulina* species

**DOI:** 10.1186/s12862-020-01617-4

**Published:** 2020-04-29

**Authors:** Chen Feng, Huiqin Yi, Lihua Yang, Ming Kang

**Affiliations:** 1 Key Laboratory of Plant Resources Conservation and Sustainable Utilization, South China Botanical Garden, Chinese Academy of Sciences, Guangzhou, 510650 China; 2 Center of Conservation Biology, Core Botanical Gardens, Chinese Academy of Sciences, Guangzhou, 510650 China; 3 University of Chinese Academy of Sciences, Beijing, 100049 China

**Keywords:** Genetic architecture, Reproductive isolation, Hybrid male sterility, QTL, *Primulina*

## Abstract

**Background:**

Sympatric sister species provide an opportunity to investigate the genetic mechanisms and evolutionary forces that maintain species boundaries. The persistence of morphologically and genetically distinct populations in sympatry can only occur if some degree of reproductive isolation exists. A pair of sympatric sister species of *Primulina* (*P. depressa* and *P. danxiaensis*) was used to explore the genetic architecture of hybrid male sterility.

**Results:**

We mapped one major- and seven minor-effect quantitative trait loci (QTLs) that underlie pollen fertility rate (PFR). These loci jointly explained 55.4% of the phenotypic variation in the F_2_ population. A Bateson–Dobzhansky–Muller (BDM) model involving three loci was observed in this system. We found genotypic correlations between hybrid male sterility and flower morphology, consistent with the weak but significant phenotypic correlations between PFR and floral traits.

**Conclusions:**

Hybrid male sterility in *Primulina* is controlled by a polygenic genetic basis with a complex pattern. The genetic incompatibility involves a three-locus BDM model. Hybrid male sterility is genetically correlated with floral morphology and divergence hitchhiking may occur between them.

## Background

Barriers to genetic exchange between diverging populations promote reproductive isolation and, ultimately, speciation. Genetic divergence of barriers between populations involves the evolution of prezygotic and postzygotic isolation [1–3]. Individual isolation barriers may be incomplete, while sequentially acting barriers restrict gene flow between diverging lineages. Reproductive isolation components that act early are often thought to contribute more to total isolation than late-acting postzygotic barriers [4, 5]. However, postzygotic isolation is undoubtedly a major factor in population divergence in the majority of systems [6]. Hybrid sterility and inviability are common forms of intrinsic barriers of postzygotic isolation, which lead to genetic differentiation and maintain species integrity [7–9].

It has been hypothesized that postzygotic reproductive isolation accumulates via increasing genomic incompatibilities [10–12], and that it is the by-product of genomic divergence [13]. Various genetic mechanisms of hybrid male sterility were proposed [14]. Of them, the Bateson–Dobzhansky–Muller (BDM) model predicts that hybrid sterility is caused by the accumulative negative interaction of two or more loci. For example, a single pair of heterospecific loci results in nearly complete hybrid male sterility in *Mimulus* [15]. Cytoplasmic male sterility (CMS) is another common genic incompatibility mechanism, which results from a mitochondrial–nuclear mismatch. The hybrid CMS mechanism has been widely studied in model species such as rice [16] and Arabidopsis [17]. In rice, a few genes causing hybrid sterility have been cloned [17–19] and several hypotheses have been proposed to explain the genetic mechanisms of hybrid sterility, such as the “duplicate gametophytic lethal model” [20] and the “one-locus allelic interaction model” [21]. In contrast to these simple models, many other studies demonstrated that hybrid sterility is a complex phenotype and is controlled by complicated mechanisms involving multiple loci. For example, empirical works in *Drosophila* and mice have revealed that hybrid sterility is highly polygenic and complex [22–24]. It is likely that genetic bases of hybrid sterility are extremely diverged in different systems. With genomic data increasingly accessible for non-model organisms [25], thousands of loci are easily identified for many lineages, potentially providing unprecedented power to study genetic mechanisms of hybrid sterility.

Theoretically, if there is gene flow between diverging species, tightly linked loci have larger selection coefficients than single loci [26, 27]. This was evidenced by many empirical studies, especially on *Mimulus* [28, 29]. Furthermore, genetic linkage will be increased between loci involved in adaptation and reproductive isolation [30–32]. Thus, a pleiotropic locus or tightly physically linked loci for adaptive traits and reproductive isolation would be the most probable way for reproductive isolation to arise. In plants, hybrid male sterility often occurs concomitantly with pleiotropic effects on floral morphology (reviewed in [33]). In *Mimulus*, hybrid male fertility is significantly correlated to corolla size phenotypically [34, 35]. In addition to pleiotropy, local adapted adjacent populations may also become reproductively isolated through hitchhiking of ecological adaptation genes and hybrid incompatibility loci [36]. Quantitative trait locus (QTL) mapping is an efficient approach for detecting pleiotropic or tightly linked loci underlying phenotypically correlated traits, and it is one of the most important fundamental methods for further detecting the hitchhiking effect.

In this study, we focus on *Primulina depressa* and *Primulina danxiaensis*, a pair of sympatric sister species (2*n* = 2*x* = 36). *P. depressa* is characterized as large and blue-purple flowers [37] while *P. danxiaensis* flowers are small and light-yellow [38], and before recent phylogenetic analysis, they were placed in two different genera [39]. Recent molecular dating revealed these two species diverged approximately 2.1 million years ago [40]. Our previous study [41] of this species pair found that flower morphological traits, as prepollination barriers, are likely subject to divergent selection. However, several lines of evidence suggest that postpollination isolation barriers are likely to play an important role in blocking hybridization between them, because of their sympatric distribution with overlapping flowering time and shared pollinators such as *Amegilla* spp., *Bombus* spp., and *Nomia* spp.. In our crossing experiments, hybrids of these sympatric sister species showed decreased male fertility. This indicats the potential postzygotic isolation barriers between *P. depressa* and *P. danxiaensis*, and makes them an ideal system for investigation of the hybrid sterility mechanisms and their evolution.

Here, we estimated hybrid male sterility in the species divergence between these sympatric sister species through heterosis analysis. We conducted a QTL mapping analysis for hybrid pollen fertility with an F_2_ population. We characterized the number, mode of action, digenic interactions, and phenotypic effects of loci that cause male sterility in F_2_ hybrids*.* We then detected whether hybrid male sterility QTLs were coincident with other flower and leaf trait QTLs. Our main objectives are to dissect the genetic basis of hybrid fertility, which may play an important role in reproductive isolation in this system.

## Results

### Analysis of variance and correlations

**Fig. 1:**
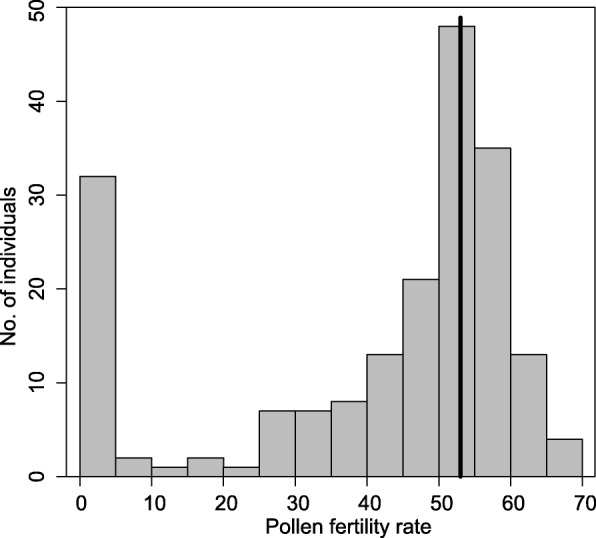
Histogram of PFR in the F_2_ population. Black vertical line indicates pollen fertility of F_1_ hybrids

The proportion of fertile and sterile pollen in all individuals was evaluated by testing pollen viability with 2,3,5-triphenyltetrazolium chloride (TTC). Under normal cultivation conditions (excluding harsh environments such as drought, flood, and extremely high or low temperatures), the pollen of parental lines (*P. depressa* and *P. danxiaensis*) was completely fertile (the pollen fertility rate [PFR] was about 95%), and the pollen of F_1_ hybrids was semi-fertile (53.02%). The PFR of F_2_ individuals ranged from 0% (completely sterile) to 69.69%, with an average of 41.17% (Fig. 1). In the F_2_ population, the PFR exhibited continuous variation, with a novel class of individuals (15.4%) that produced no viable pollen grains (Fig. 1).

**Fig. 2:**
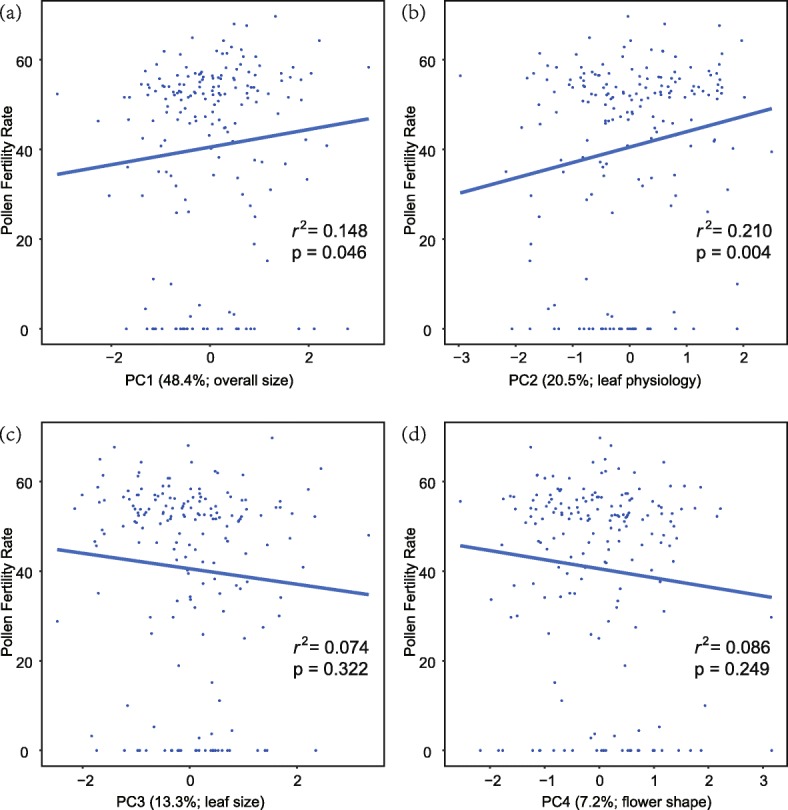
Scatter plots showing the correlation between PFR and PCs derived from morphological and physiological traits. Percentages of total variation for each PC explained and interpretation for each PC represented were indicated in brackets

In F_2_ hybrids, PC1 and PC2, which represent variation in plant overall size and leaf physiology, showed weak positive but significant correlations with PFR (Fig. 2). For individual traits, corolla length, stamen length, and leaf pigment concentration traits were significantly correlated with PFR (Additional file 1: Table S1).

### Mid-parent heterosis in 13 traits

Mid-parent heterosis (MPH) was estimated as the percentage of the difference between the mean of F_1_ or F_2_ and the mid-parent mean divided by the mid-parent mean. We estimated MPH for PFR and 12 flower and leaf traits that we studied previously [41]. PFR displayed almost the lowest MPH among all of the traits (Table 1). MPH for PFR in F_1_ was − 44.5%. The performance of 12 flower and leaf traits for F_1_ hybrids, the F_2_ population, and their parental lines is listed in our previously published paper [41]. In these traits, the concentration of leaf pigments showed the highest MPH (an average of 31.9%) in F_1_ (Table 1).

**Table 1:** Mid-parent heterosis (MPH) for PFR and 12 flower and leaf traits in our previous study. If %MPH > 0, this represents the percentage of individuals showing MPH > 0 in all of the measured F_2_ individuals

Trait	Unit	MPH of F_1_	MPH of F_2_			%MPH > 0
			Mean ± SD	Max	Min	
Pollen fertility rate	%	−44.5%	−0.469 ± 32.58	−0.047	− 1.000	0.00
Upper petal width	mm	7.4%	−0.103 ± 13.21	0.294	−0.412	18.95
Lower petal width	mm	1.2%	−0.111 ± 11.81	0.231	−0.438	20.53
Corolla width	mm	5.0%	−0.053 ± 12.80	0.479	−0.339	32.63
Corolla height	mm	18.6%	−0.060 ± 11.94	0.330	−0.407	35.26
Corolla length	mm	−41.2%	−0.007 ± 13.94	0.323	−0.472	47.37
Pistil length	mm	4.2%	0.003 ± 10.87	0.284	−0.245	50.00
Stamen length	mm	−0.2%	−0.002 ± 10.63	0.310	−0.288	51.31
Maximum leaf length	cm	−36.0%	−0.389 ± 24.89	0.019	−0.697	0.52
Maximum leaf width	cm	−7.8%	−0.120 ± 25.91	0.466	−0.559	17.28
Chlorophyll a concentration	mg/m^2^	36.4%	0.559 ± 11.75	1.192	−0.050	98.97
Chlorophyll b concentration	mg/m^2^	35.1%	0.484 ± 19.23	1.116	−0.188	97.42
Carotenoid concentration	mg/m^2^	24.2%	0.496 ± 11.90	1.103	−0.113	98.97

Considerable transgressive segregation was observed for PFR and leaf pigments in F_2_ individuals (Fig. 1; Table 1; data from [41]). As described above, all of the F_2_ individuals produced less fertile pollen than parental lines. Almost all of the F_2_ individuals showed heterosis in leaf pigments (Table 1).

### QTL analyses

**Fig. 3:**
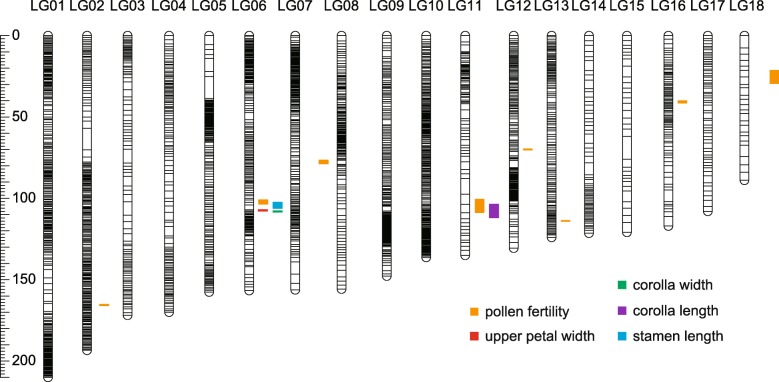
Genome location of significant PFR QTLs and two co-localized floral morphology QTLs. Confidence intervals of QTLs are illustrated on the right side of linkage groups

**Fig. 4:**
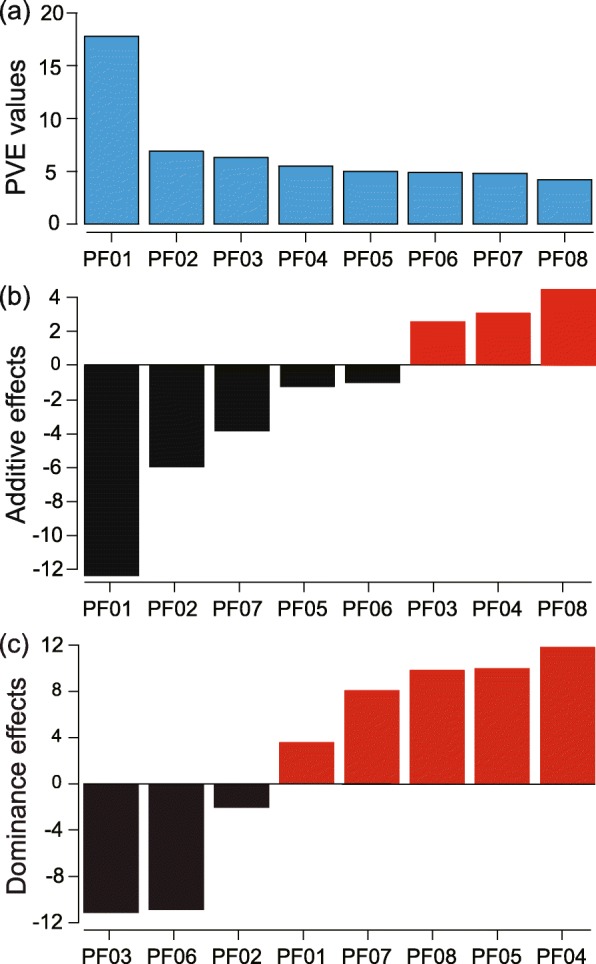
The effect size, additive effect distribution, and dominance effect distribution of QTLs for hybrid PFR are shown in (**a**), (**b**), and (**c**). The *x*-axis indicates the name of each QTL. The *y*-axis in **a** indicates the phenotypic variation explained by each QTL (PVE). The *y*-axis in **b** and **c** indicates the additive and dominance effects of the *P. depressa* allele relative to the *P. danxiaensis* allele at each QTL

The linkage map and QTL mapping analysis for floral and leaf traits were described in our previous study [41]. We detected QTL for PFR here with MapQTL v.6.0 software using 1 cM increments [42]. Table 2 summarizes QTL mapping results for PFR, and Fig. 3 shows locations of QTLs. A total of eight QTLs were identified for PFR, which jointly explained 55.4% of the phenotypic variation in the F_2_ population (Table 2). The phenotypic variation explained by each QTL ranged from 4.2% (PF02) to 17.8% (PF01). In particular, PF01 on LG02 is a major-effect locus for PFR, with an LOD value of 12.09. The *P. depressa* allele at PF01 resulted in 12.44% less pollen viability. Except PF01, all QTLs explained less than 7% of the total phenotypic variation (Fig. 4a and Table 2). These results suggest that the genetic architecture of pollen fertility contains a major-effect QTL plus numerous minor-effect QTLs.

**Table 2:** Summary of QTLs detected for PFR and overlapping loci for two floral morphological traits

Locus ID	LG	Position (cM)	Nearest RAD locus	LOD	1.5 LOD unit of support (range in cM)	% Expl.	QTL direction
PF01	2	165.8	152,874	12.09	165.19–165.78	17.8	–
PF02	6	102.4	170,253	3.18	101.00–103.36	4.2	–
PF03	7	77.6	19,822	5.11	76.63–78.69	6.9	+
PF04	11	105.7	152,571	4.18	100.53–108.68	5.5	+
PF05	12	70.3	42,292	3.76	69.48–70.27	5.0	–
PF06	13	114.2	117,627	4.67	113.51–114.19	6.3	–
PF07	16	40.8	60,459	3.66	40.01–41.36	4.9	–
PF08	18	27.4	114,119	3.63	21.30–29.35	4.8	+
SL03	6	103.362	33,528	4.34	102.362–105.998	5.3	+
UPW06	6	107.81	94,126	5.9	106.95–107.81	7.6	+
CW02	6	107.81	101,936	5.11	107.81–108.37	7.7	+
CL07	11	107.676	124,189	3.74	103.676–111.772	4.4	+

LG refers to the linkage group with which the QTL was detected. QTL interval and position are given in centimorgan (cM). The LOD significance level was determined by 10,000 permutations. % Expl. represents the phenotypic variation effect. In the QTL direction column, +/− indicates QTL effects are/are not in the direction of trait divergence between parental species. All of the QTLs were found at the 0.5% significance level

*PF* pollen fertility, *SL* stamen length, *UPW* upper petal width, *CW* corolla width, *CL* corolla length

**Fig. 5:**
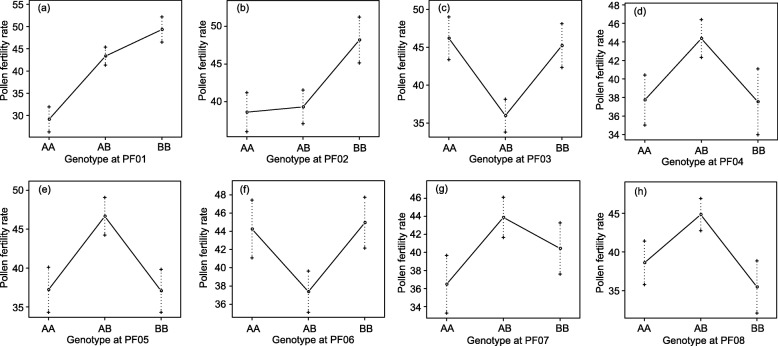
Phenotypic trait means of different genotypes of each QTL

For five of the eight pollen fertility QTLs (PF01, PF02, PF05, PF06, and PF07), *P. danxiaensis* alleles were associated with decreases in pollen viability (Table 2 and Figs. 4b and 5). Analysis of the dominance effect showed that three QTLs (PF02, PF03, and PF06) were dominant with *P. danxiaensis* alleles, and the other five were dominant with *P. depressa* alleles (Table 2 and Figs. 4c and 5).

Comparing with previous QTLs for 12 flower and leaf traits for the same population, we found two QTLs for pollen fertility (PF02 and PF04) overlapped with QTLs for floral morphology (corolla length and stamen length) (Table 2 and Fig. 3). We calculated the significance of correspondence between these overlapping QTLs and found that both pairs showed significantly more overlap than expected by chance (*P* < 0.05). In addition, we found two additional floral morphology QTLs (upper petal width and corolla width) were tightly linked (about 5 cM) with PF02 (Table 2 and Fig. 3). As we previously found that divergence in flower morphology was driven by natural selection [41], there was probably a hitchhiking effect between hybrid sterility and floral traits. However, we did not find the evidence of QTL overlap between PFR and leaf physiology.

### Gene action and locus interaction

According to gene action estimation, two of the eight identified QTLs (PF01 and PF02) exhibited partial dominance, and the other six exhibited overdominance (Table 3). For the six overdominant loci, heterozygous genotypes reduced the pollen fertility at loci of PF03 and PF06 (Table 3 and Fig. 5). The underdominance evidenced the conflict between parental alleles. Conversely, the heterozygous genotype was favorable for pollen fertility at four loci (PF04, PF05, PF07, and PF08; Table 3 and Fig. 5). The overdominance suggests that heterosis occurs at some loci.

**Table 3:** The degree of dominance was calculated as dominance effect/|additive effect|

Loci	Additive effect	Dominance effect	Degree of dominance	GA	Mean AA phenotype	Mean AB phenotype	Mean BB phenotype	Epistatic interaction loci	Segregation distortion	
									*P*-value	Significance
PF01	−12.44	3.58	0.29	PD	20.58	36.61	45.47		0.802	–
PF02	−5.98	−1.99	−0.33	PD	27.05	31.03	39.00	PF08	0.019	*
PF03	2.48	−11.06	−4.46	OD	35.47	22.22	30.58	PF08	0.355	–
PF04	3.00	11.80	3.93	OD	35.07	42.84	30.99		0.089	–
PF05	−1.21	9.97	8.22	OD	31.81	43.00	34.24		0.013	*
PF06	−0.95	−10.83	−11.34	OD	32.07	22.20	33.98		0.087	–
PF07	−3.83	8.06	2.10	OD	29.20	41.09	36.86		0.447	–
PF08	4.46	9.80	2.20	OD	35.94	41.06	30.11	PF02; PF03	0.439	–

GA shows gene action modes, which are classified as follows: A, additive (|d/a| ≤ 0.20); PD, partial dominance (0.20 < |d/a| ≤ 0.80); D, dominance (0.80 < |d/a| ≤ 1.20); and OD, overdominance (|d/a| > 1.20). Mean phenotype traits of the *P. depressa* homozygote (AA), the heterozygote (AB), and the *P. danxiaensis* homozygote (BB) are shown. The *P*-value and significance of each locus segregation distortion were calculated

* *P* < 0.05

**Fig. 6:**
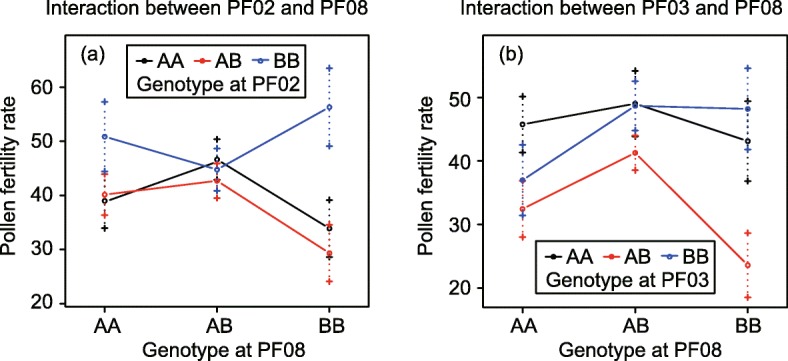
Digenic interactions between two pairs of QTLs. AA, BB, and AB represent homozygous genotypes of male and female parents and a heterozygous genotype

We characterized pairwise QTL interactions to summarize epistatic interactions at a threshold of *P* < 0.05 (Additional file 2: Table S2). Digenic interactions were identified between two QTL pairs: PF02/PF08 and PF03/PF08 (Fig. 6; Tables 3; Additional file 2: Table S2). A high level of male sterility occurred in individuals that harbored alleles from different parents at PF03 and PF08 simultaneously (e.g., heterozygous alleles or homozygous *P. danxiaensis* alleles at PF03 and homozygous *P. depressa* alleles at PF08). A similar pattern was observed between PF02 and PF08; lower PFR occurred in individuals that were homozygous for *P. danxiaensis* alleles at PF08 and homozygous for *P. depressa* alleles or heterozygous for both parental alleles at PF02.

The estimation of locus segregation distortion revealed that peak LOD markers and their surrounding markers of PF02 distorted to the male parent (*P* < 0.05), while PF05 distorted to the female parent (Tables 3; Additional file 3: Table S3). Peak LOD markers and many surrounding markers of PF04, PF07 and PF08 distorted to female and male parents, respectively, even though not significantly at peak LOD markers (Additional file 3: Table S3).

## Discussion

### Hybrid inviability and sterility

Studying mechanisms of reproductive isolation barriers is important for improving our understanding of the process of speciation [3, 5]. We identified multiple potential prezygotic reproductive isolation barriers that could contribute to the divergence between sympatric *P. depressa* and *P. danxiaensis* [41]. However, the incomplete pollinator divergence and the overlapping flowering time indicate that the prezygotic reproductive isolation might be insufficient for blocking the hybridization between *P. depressa* and *P. danxiaensis*. In this study, approximately one-sixth of the F_2_ hybrids showed complete male sterility (Fig. 1), which indicates hybrid male sterility has contributed, at least partially, to reproductive isolation between these two species.

Hybrid inviability and sterility are two of the most common forms of postzygotic isolation in plants, because they prevent gene flow between lineages. In our case, we studied MPH for hybrid fertility and 12 flower and leaf traits to unravel the mechanism of postzygotic isolation between *P. depressa* and *P. danxiaensis* in sympatry. In F_1_ hybrids, pollen fertility showed a greater underdominance than all of the flower and leaf traits investigated (Table 1), suggesting that male sterility plays an important role in postzygotic reproductive isolation in this case. F_1_ hybrids showed high concentrations of chlorophylls which represent potentially better photosynthesis capacities, but they did not show significant heterosis or depression for most of the traits for floral morphology and leaf size (abstract values of MPH were less than 10%; Table 1). Considerable transgressive segregation of leaf pigments in F_2_ population indicated that the photosynthetic capacity of most F_2_ hybrids was better than the parental lines (Table 1; data from [41]). This heterosis for photosynthesis was consistent with the action of loci in leaf pigments, 27 out of 30 loci were dominant or overdominant [41]. MPH value of all F_2_ individuals for PFR were negative, and this was possibly caused by genetic conflict (Table 1). Collectively, these results indicate hybrid sterility might play an important role in postzygotic reproductive isolation, while we did not observe apparent hybrid inviability, at least in our greenhouse. Further study of the relative importance of hybrid male sterility and hybrid inviability will be needed in the future.

Artificial crossing between *P. depressa* and *P. danxiaensis* produced vigorous but semi-sterile hybrids. Most *Primulina* species are narrow endemics with small population sizes [43]. Small-size populations usually have lower genetic diversity and are prone to accumulating deleterious recessive alleles [44–47]. If chlorophyll content and photosynthetic capacity are maladaptive in this system, it will make sense that genetic incompatibilities decrease fertility but increase vigor in interspecific hybrids here. Similar conclusions have also been drawn in many other plant species, such as *Luffa* [48] and rice [49–52].

### Genetic architecture of hybrid male sterility

We found a polygenic basis for PFR in *P. depressa* × *P. danxiaensis* F_2_ hybrids. We identified one major-effect QTL, which explained 17.8% of the hybrid population phenotypic variation. Seven minor-effect individual QTLs explained 4.2–6.9% of the phenotypic variance (Table 2). These QTLs jointly accounted for 55.4% of the total phenotypic variation in hybrid pollen fertility. Even though a low number of QTLs may have remained hidden due to the modest size of the mapping population (*N* = 195), the genetic architecture of postzygotic isolation in our system was polygenic, as we found a major-effect QTL plus numerous minor-effect QTLs.

The polygenic genetic basis of hybrid male fertility is consistent with that observed in other plant species. For example, eight-QTL hybrid sterility was identified between two closely related species of *Solanum* [53], and a number of different QTLs for male sterility were mapped in two subsequent mapping experiments [54, 55]. Likewise, studies in *Drosophila* indicated that interspecific male and female sterility were highly polygenic (e.g., [56, 57]). Tao et al. [22] identified a total of approximately 60 QTLs contributing to hybrid male sterility when *Drosophila mauritiana* is introgressed into a *Drosophila simulans* background, indicating that a polygenic genetic basis of hybrid male sterility is prevalent in both plants and animals. However, two-locus genetic incompatibility was reported to cause male sterility between species or subspecies in rice [58, 59] and *Mimulus* [15]. Lowry et al. [60] detected three sterility loci in hybrids of two *Panicum hallii* ecotypes. In general, fewer loci contribute to hybrid sterility in plants than in *Drosophila*, perhaps because plants largely lack sex chromosomes [56, 61].

In our *Primulina* study, the major-effect QTL contributes nearly 18% of the hybrid male sterility. This large effect makes it possible that divergence of this QTL is sufficient to impede gene flow and contribute to species integrity in the presence of vigorous hybrids. According to mean phenotype values at this major-effect locus (PF01), homologous *P. danxiaensis* alleles increase pollen fertility two-fold compared with homologous *P. depressa* alleles. Because mitochondrial genomes of hybrids were inherited maternally from *P. danxiaensis*, we suspect that CMS is a possible mechanism underlying hybrid male sterility here. Further elucidation of the molecular basis of PF01 will provide insight into its normal function within pure species.

The QTL overlap between PFR and flower morphology (corolla length and stamen length) suggests that hybrid male sterility is genetically correlated with floral morphological traits (Fig. 3 and data from [41]). In addition, a PFR QTL (PF02) was tightly linked to upper petal width and corolla width QTLs (Fig. 3 and data from [41]). This genotypic correlation pattern, including pleiotropic and tightly linked loci, was consistent with our phenotypic correlation analysis (Fig. 2; Additional file 1: Table S1). When a favorable mutation arises, the allele frequency of closely linked surrounding loci will also increase (i.e., genetic hitchhiking) [62]. Inversely, deleterious mutations will also eliminate the variation at their surrounding loci (i.e., background selection) [63]. In our case, flower morphology was divergently selected [41], and it may affect selection on hybrid male sterility genes when hitchhiking occurs. In consideration of both QTL overlap and close linkage of male sterility and morphological traits, we suspect that hybrid sterility evolved as an incidental by-product of hitchhiking. Groups of tightly linked minor-effect loci have larger selection coefficients than single loci with small phenotypic effects [25]. Therefore, a single locus that affects both adaptive and reproductive isolation traits would be the most probable way for selection to maintain differentially adapted species, even in the face of gene flow. These results are consistent with recent studies on *Mimulus* (e.g. [28, 29, 64]).

The coincidental (phenotypic and genotypic) correlation between PFR and flower morphology suggests developmental integration. In accordance with our results, a recent study in *Mimulus* found a genetic correlation between flower size and hybrid male fertility [35]. The inconsistent directionality of QTL effects suggests that reduced flower size in hybrids increases male fertility. In consideration of the association between self-fertilization and reduced flower size [64], small flowers theoretically suppressed gene exchange between *P. depressa* and *P. danxiaensis* yet promote hybrid male fertility. However, further crossing experiments for estimating outcrossing rates are necessary to evidence the correlation between gene exchanging rate, flower size, and hybrid fertility.

Leaf pigments play important roles in responses to biotic and abiotic stresses and thus are sensitive to environmental changes [65–67]. Pollen fertility is easily affected by environmental factors as well [18, 19, 68]. Drought stress, a common instance of environmental stress, was reported to decrease both pollen fertility [69] and leaf chlorophyll content [70, 71] in wheat. This is one of the probable reason why, in our study, pollen fertility was correlated with leaf pigments phenotypically but not genotypically. Further work is needed to test the relationships among stresses, leaf pigments and pollen fertility.

### Complexity of hybrid sterility

Significant negative epistatic interactions were observed between two pairs of QTLs: PF02/PF08 and PF03/PF08 (Fig. 6; Table 3; Additional file 2: Table S2). This indicates that the BDM model involving three loci contributes to hybrid male sterility. According to the BDM model, sterility is more polymorphic within plant species than animal species (reviewed in [61]). The polygenic BDM model in our case implies the sterility may be under complex genetic control.

Besides the BDM model, segregation distortion is very common at loci surrounding hybrid male fertility QTLs in our study. Two QTLs (PF02 and PF05) showed significant segregation distortion at their peak LOD and surrounding loci (Additional file 3: Table S3). PF04, PF07, and PF08 did not show significant segregation distortion at peak LOD loci, but their surrounding loci did (Additional file 3: Table S3). The segregation distortion may resulted from gametes abortion (prezygotic selection) or the selective fertilization of particular genotypes (postzygotic selection). Unfortunately, we couldn’t distinguish them from a single F_2_ linkage map (reviewed by [72, 73]). Correlations between locus segregation distortion and male sterility has been reported in a few systems, including *Drosophila* (e.g., [74–76]). In rice, segregation distortion sometimes has significant effects on hybrid male sterility [77–79]. To our knowledge, however, this is the first study on correlation between locus segregation distortion and hybrid male fertility in non-model plants.

In contrast to *Mimulus* species [15], we here show a complex genetic basis for hybrid male sterility between *P. depressa* and *P. danxiaensis*. Hybrid male sterility between *Mimulus guttatus* and *Mimulus nasutus* were almost results from a simple genetic incompatibility between a single pair of heterospecific loci. In contrast, our results show a complex genetic basis of hybrid male sterility, including a BDM model involving three loci and genetic correlations with flower morphology. Further genetic dissection may reveal that more complicated mechanisms underling each locus. A more complex genetic architecture of a reproductive isolation barrier will reduce the rate of gene flow across the entire genome to a greater extent than a barrier governed by only a few major loci [6]. Therefore, the identified complex genetic architecture of hybrid male sterility might make a critical contribution to the maintainance of species identity between *P. depressa* and *P. danxiaensis*.

## Conclusions

In this study, we found that hybrid male sterility in *Primulina* is controlled by a polygenic genetic basis. The genetic incompatibility involves a three-locus BDM model, even though the single largest-effect locus is not involved in. Hybrid male sterility is genetically correlated with floral morphology and divergence hitchhiking may have occurred between them. This complex pattern is in contrast to the simple genetic incompatibility model found in *Mimulus* [15]. The identified complex genetic architecture of hybrid male sterility might play an important role in maintaining species identity between *P. depressa* and *P. danxiaensis*. Continued studies of reciprocal backcross can provide detailed incompatibility patterns and further our understanding of evolutionary forces driving postzygotic reproductive isolation in this system.

## Methods

### Plant materials and growth conditions

We collected parental lines (*P. depressa* and *P. danxiaensis*) at the sympatric site of Danxia Mountain, Guangdong, South China. With the permission of Danxia Mountain National Park Commission, all samples were originally collected following methods that met the guidelines of Regulations on Wild Plants Protection (People’s Republic of China) for the use of plants in research. Voucher specimens of *P. depressa* (*DXS02*) and *P. danxiaensis* (*DXS04*) were deposited in the South China Botanical Garden Herbarium (IBSC). We created a F_2_ mapping population, including 201 individuals, by self-fertilizing one F_1_ plant derived from a cross between an individual of *P. depressa* (♂) and an individual of *P. danxiaensis* (♀). F_1_ hybrids, male and female parents, and 201 F_2_ individuals (the mapping population) were genotyped by restriction site-associated DNA sequencing (RAD-seq). A high-density linkage map was constructed with 2484 markers that were spaced across 18 linkage groups. For further details, see [41].

To determine male fertility, we planted parental populations, F_1_ hybrids, and the F_2_ population in a greenhouse in South China Botanical Garden under fluorescent light to provide a 14-h day length, with a temperature of ~ 26 °C. Plants were watered by sub-irrigation as needed and fertilized weekly.

### Male fertility assessment and pollen staining

Pollen viability was assessed by staining pollens with TTC [80]. For pollen staining, we collected the first three flowers per individual on the day the flower opened. These flowers contained mature pollen, and there was no bias in the analysis of each individual. We halved anthers with a pair of forceps, and then pollen was squeezed into 50 μl of TTC (1% w/v in 50% sucrose) solution on a glass slide and incubated in the dark at room temperature for 15 min. TTC white compound can be enzymatically converted to red 1,3,5-triphenylformazan (TPF) by various dehydrogenases in metabolically active pollen. Therefore, red stained pollen grains were considered viable, while faintly stained or empty pollen were non-viable. More than 100 pollen grains were observed for each flower under the microscope. The percentage of viable pollens in the total examined pollens was calculated as the pollen fertility rate (PFR).

### Phenotypic data analysis

We previously performed principal component analysis (PCA) on seven floral traits (upper petal width, lower petal width, corolla width, corolla height, corolla length, pistil length and stamen length), three leaf physiological traits (concentrations of chlorophyll a, chlorophyll b, and carotenoids), and two morphological traits (leaf length and leaf width). The first two principal components (PCs) accounted for 48.4 and 20.5% of the total measured phenotypic variation, with the first component correlating with overall size (in particular the size of floral traits) and the second with leaf physiology traits. For detail information, see [41]. Here, we calculated Pearson’s correlation coefficients between PFR and the 12 previously studied traits and PCs derived from them.

In order to evaluate the hybrid phenotypic performances, a previously developed mid-parent heterosis (MPH) statistical test [81, 82] was used. MPH was calculated using the following formula:

$$ \mathrm{MPH}=\frac{\mathrm{Hybrid}\ \mathrm{mean}-\mathrm{Mid}-\mathrm{parent}\ \mathrm{mean}}{\mathrm{Mid}-\mathrm{parent}\ \mathrm{mean}}\times 100\%, $$

Where mid-parent mean represents the average of both parents with respect to a trait of interest. In this study, we performed MPH analysis on 13 phenotypic traits mentioned above.

### QTL mapping

The linkage map used in this study was a highly resolved RAD-seq-based SNP map with 2484 markers distributed across the 18 linkage groups with an average distance of 0.96 cM [41]. The QTL intervals of PFR were defined on the linkage map with MapQTL v.6.0 software [42]. Before QTL mapping, a genome-wide and chromosome-level LOD score threshold (*P* < 0.05) for declaring the presence of QTLs was determined using the permutation test (10,000 replications). Based on the permutation results, an LOD score threshold of 3.0 was set for the trait to declare the presence of a significant QTL. The potential QTLs were initially detected employing the Interval Mapping (IM) algorithm. Then, markers with the highest LOD values were selected as cofactors, and the final set of markers detected at *P* < 0.05 after automatic cofactor selection were further included in the Multiple QTL Model (MQM). Cofactor selection and MQM analysis were repeated until the best possible set of QTLs was found, and then each QTL was characterized by its maximal LOD score. The 1.5-LOD support intervals were estimated in centimorgans (cM) all of the significant QTLs detected. Final QTL maps were drawn with the help of the graphical package MapChart v2.2 [83].

To evaluate the significance of correspondence between QTL and PFR or other traits that we studied previously [41], we calculated the probability of obtaining the observed number of matching QTLs by chance alone [84]. This probability was estimated using a hypergeometric probability distribution function [85]. The equation used is shown in [56].

### Gene action and locus interaction analysis

Gene action was proposed to estimate the relative importance of the dominance effect and the additive effect, and it was estimated by calculating |d/a| = |dominance effects/additive effects| [86]. Results were defined as follows: A, additive (|d/a| ≤ 0.20); PD, partial dominance (0.20 < |d/a| ≤ 0.80); D, dominance (0.80 < |d/a| ≤ 1.20); and OD, overdominance (|d/a| > 1.20). Thresholds were firstly proposed by [86], and commonly used in many following studies (e.g., [87–89]).

Digenic epistatic interactions were analyzed with markers closer to QTL peaks using R/qtl [90]. The proportion of variance explained by epistasis was tested by comparing the residual of the full model containing all of the single-locus effects and two-locus interaction effects with that of the reduced model containing all of the single-locus effects but excluding two-locus interaction effects.

In addition to gene action and digenic interactions, segregation distortion of mapped QTLs and their closely linked markers was estimated. The degree of marker segregation distortion in the F_2_ generation was determined by marker data comparison against the expected 1:2:1 ratio using the Chi square test, where significant distortion was declared at *P* < 0.05.

## Supplementary information

**Additional file 1 **: **Table S1.** Linear correlation (Pearson) among the previously studied 12 flower and leaf traits and pollen fertility rate in F_2_ population. Values in bold are significant (*p* < 0.05).

**Additional file 2 **: **Table S2.** Summary of significant epistatic interactions between QTLs. Epistasis analyses among the identified QTLs were conducted with R/qtl. Var (%), *F*-value, and *P*-value represent percentage of variance explained, *F* statistics, and *F* distribution, respectively. * *P* < 0.05, ** *P* < 0.01.

**Additional file 3 **: **Table S3.** Genotype distribution of peak LOD loci of QTLs and their surrounding ten loci. The Chi square test was used. * *P* < 0.05, ** *P* < 0.01. Peak LOD loci of each QTL are presented in bold.

## Data Availability

Pollen fertility rate data for QTL analysis are available at the FIGSHARE repository: DOI: 10.6084/m9.figshare.8949218.

## Abbreviations

BDM: Bateson–Dobzhansky–Muller
CMS: Cytoplasmic male sterility
PFR: Pollen fertility rate
QTL: Quantitative trait loci
RAD: Restriction site-associated DNA
